# Supplementary material for: Development of a Self-Care Scale for Compound Caregivers
Source: Healthcare (Basel). 2024 Dec 4;12(23):2438. doi: 10.3390/healthcare12232438 (PMC11640845; doi:10.3390/healthcare12232438)
Supplement: Supplementary file 1 [file healthcare-12-02438-s001.zip › Supplementary Material S1 Japanese version of the SCSCC.pdf]

多重介護者におけるセルフケア尺度

多重介護を担うご家族が行っている“ご自身”の健康管理についてお尋ねします。
各項目の活動について、あなたはどのくらいの頻度でありますか。
それぞれ最もあてはまるものを丸（〇）で囲んでください。

№	項目	全くしていない	あまりしていない	している	とてもよくしている
1	定期的に気分転換の時間を確保する	0	1	2	3
2	介護を頑張っている自分を、<br>自分なりにほめてあげている	0	1	2	3
3	自分の不調に気づいてくれる、<br>友人やピアサポーターにつながる	0	1	2	3
4	自身の限界を知る	0	1	2	3
5	自分の溜め込んでいるストレスに<br>気づくことができる	0	1	2	3
6	自身の健康の維持について、<br>だれかに助言をしてもらえる環境を<br>確保している	0	1	2	3
7	人に頼めることは、<br>自分で抱え込まずにだれかに<br>依頼している	0	1	2	3
8	ケアや日常生活援助のサービスを<br>利用している	0	1	2	3

合計得点範囲：最低 0～最高 24 点

出典）Iwata Y., Minamizaki M. and Kanoya Y. Development of a self-care scale for compound caregivers. Healthcare. 2024
