# Supplementary material for: Development of a Self-Care Scale for Compound Caregivers
Source: Healthcare (Basel). 2024 Dec 4;12(23):2438. doi: 10.3390/healthcare12232438 (PMC11640845; doi:10.3390/healthcare12232438)
Supplement: Supplementary file 1 [file healthcare-12-02438-s001.zip › Supplementary Material S2 English version of the SCSCC.pdf]

The Self Care Scale for Compound Caregivers (SCSCC)

We would like to inquire family members with compound caregiving responsibilities about their own health management.
How often do you engage in the activities listed below? Please select the most relevant option by circling it.

No	Item	Not at all	Not much	Some times	Very often
1	I ensure to take time to refresh myself regularly	0	1	2	3
2	I acknowledge my effort as a caregiver	0	1	2	3
3	I connect with friends or peer supporters who can recognize when I am not feeling well	0	1	2	3
4	I know my limits	0	1	2	3
5	I recognize the stress I have accumulated	0	1	2	3
6	I have access to advice on maintaining my health	0	1	2	3
7	I delegate tasks instead of taking on everything myself	0	1	2	3
8	I utilize care and daily life support services	0	1	2	3

The total score range is from 0 to 24 points.

Iwata Y., Minamizaki M. and Kanoya Y. Development of a self-care scale for compound caregivers. Healthcare. 2024
